# On the Development of Innovative Column Internals for Nitrogen Recovery from Agricultural Waste

**DOI:** 10.1021/acsomega.5c12115

**Published:** 2026-07-02

**Authors:** Sören A. Bernemann, Jan F. Maćkowiak, Jerzy Maćkowiak, Eugeny Y. Kenig

**Affiliations:** † Chair of Fluid Process Engineering, 26578 Paderborn University, Pohlweg 55, Paderborn 33098, Germany; ‡ ENVIMAC Engineering GmbH, Im Erlengrund 27, Oberhausen 46149, Germany

## Abstract

Using agricultural waste as fertilizer results in contamination of groundwater, leading to environmental and health risks. A partial removal of ammonia is necessary before the waste application on fields, which can be realized via an air-stripping process. However, conventional column internals for air stripping are prone to clogging because of the high solid loads of the liquid waste. This was an impetus for the development of an innovative separator with new types of internal structures based on inclined plates. A numerical model and an experimental setup were established in our previous work and used to investigate the plates. In the present study, first, the initial packing concept was evaluated, showing promising results. Subsequently, the packing was optimized toward increasing the gas–liquid interfacial area, using both experimental and simulation-based approaches. The optimized internals prevent clogging, allowing sustainable continuous use of the column and making air stripping a more viable option for removing ammonia from liquid agricultural waste.

## Graphical abstract

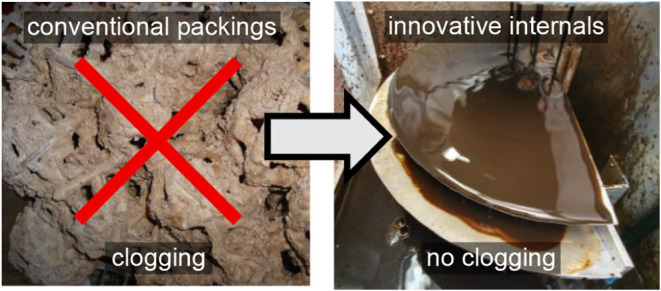

## 1 Introduction

Liquid agricultural waste from biogas plants, primarily composed of liquid manure, is widely used as a fertilizer in farming. The waste often contains significant amounts of nitrogen compounds, particularly ammonia, which can result in soil and groundwater contamination. This contamination poses environmental risks, such as eutrophication of aquatic ecosystems, and threatens human health, with nitrate-contaminated drinking water being linked to potential carcinogenic effects.^1^ A reasonable mitigation strategy to solve this problem is the removal of ammonia from liquid waste prior to its application on agricultural land. This requires a simple and cost-effective process that can be operated directly at a biogas plant or on a farm.

The literature on ammonia reduction in cattle manure and other agricultural wastes primarily addresses the mitigation of air pollution caused by gaseous ammonia emissions,^2–4^ with limited attention given to the issue of groundwater contamination. Furthermore, studies concerning the reduction of ammonia in liquids predominantly focus on industrial wastewater, with no relation to agricultural waste. Here, the treatment of wastewater with microorganisms,^5^ ammonia adsorption on composting material,^6^ and the application of hydrophobic membranes^7^ are studied. The application of air stripping for ammonia removal from liquid manure was investigated by Liao et al.^8^ They analyzed the impact of temperature, air-to-liquid flow ratio, and pH value on the removal efficiency of ammonia from swine manure. The primary objective of high removal efficiencies was achieved; however, this was accomplished using a lab column equipped with a plastic ring-packed bed which is prone to clogging due to the solid content of swine manure. Therefore, this method is not suitable for continuous operations at biogas plants. A further study on the ammonia stripping from swine manure was conducted by Bonmatí and Flotats^9^ who aimed at the complete removal of ammonia from pig slurry as a pre- or posttreatment to anaerobic digestion. Bonmatí and Flotats^9^ focused on the effects of the process parameters temperature and pH value on ammonia removal via air stripping. However, the problem of column clogging resulting from the solids of the agricultural waste was not addressed. Song et al.^10^ compared different stripping configurations covering pretreatment, post-treatment, in situ, and side-stream stripping of biogas waste regarding ammonia control and recovery effectiveness. Equipment clogging was identified as a key challenge of the stripping process. A method preventing such clogging in stripping systems is using a fluidized packed bed (FPB), as presented by Maćkowiak et al.^11^ Although air stripping is possible in FPBs, the backpressure, which is 10 to 20 times higher than in conventional stripping columns, represents a significant disadvantage. A new approach to ammonia reduction from biogas slurry is the membrane-based electrochemical ammonia stripping presented by Zhang et al.^12^ This method promises high energy and cost efficiency; however, membrane clogging poses a problem, and the concept needs further investigation beyond the lab scale. A different approach was investigated by Huang et al.^13^ by removing ammonia with microorganisms in aquatic systems. Although high removal efficiencies were achieved, this method has so far been used mainly in laboratory studies, while its industrial application is challenging.

Despite the fact that air and steam stripping processes to remove ammonia in agriculture have been used for around 30 years,^14^ the problem of column clogging using conventional internals has not been addressed. Because of the solid content of liquid agricultural waste, air stripping with conventional column internals such as packed beds cannot be used for sufficiently long durations. The high solid load of the liquid waste leads to residue buildup and the subsequent clogging of the column. To solve this problem, an innovative separator design, based on inclined plates, was proposed in our previous work.^15^ For the development of this new design, the flow behavior of the liquid agricultural waste was investigated experimentally and numerically.

In the present work, the experimental setup and the numerical simulation model presented in ref 15 were adapted and applied to investigate the impact of the physical properties of liquid agricultural waste on an innovative separator design. Furthermore, prototypes of the new internals of the separator were tested regarding the prevention of column clogging and optimized with respect to their flow and wetting behavior.

## 2 Methods

### 2.1 Materials

The agricultural waste used in our work was fermentation residues from biogas plants located in Borken and Wesel, Germany. The solid content of the waste was reduced in a preprocessing step with a screw press separator from UTS Products GmbH to a mass fraction of 5–6%. The density was measured as 1028 kg/m^3^. Viscosity was determined using a Modular Compact Rheometer (MCR501) from Anton Paar with the CC27 coaxial cylinder measurement system showing non-Newtonian behavior, most likely resulting from the fiber particles in the fluid. The measurement results are depicted in Figure 1.

**Figure 1:**
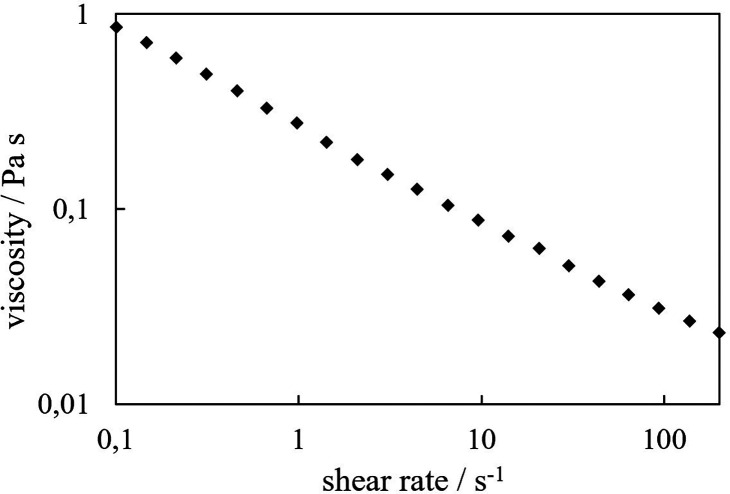
Results of viscosity measurement of the liquid agricultural waste illustrating non-Newtonian behavior.

All experimentally tested components, including inclined plates and column internal prototypes, were made from stainless steel 1.4571 and aluminum Al99.5.

### 2.2 Experiment

#### 2.2.1 Experimental Setup

The experimental setup presented in the previous publication^15^ was used and adapted for the experimental studies. It consists of a reservoir, an impeller pump, a bypass, and a liquid distributor, as shown in Figure 2a. Inside the reservoir, a test structure was placed under the liquid distributor. This structure is either a flat inclined plate (Figure 2a and Figure 3a) or a cone-and-funnel combination, hereinafter called CAF (Figures 2b and Figure 3b). The liquid flow can be observed through a window on the side of the reservoir (cf. Figure 3a).

**Figure 2:**
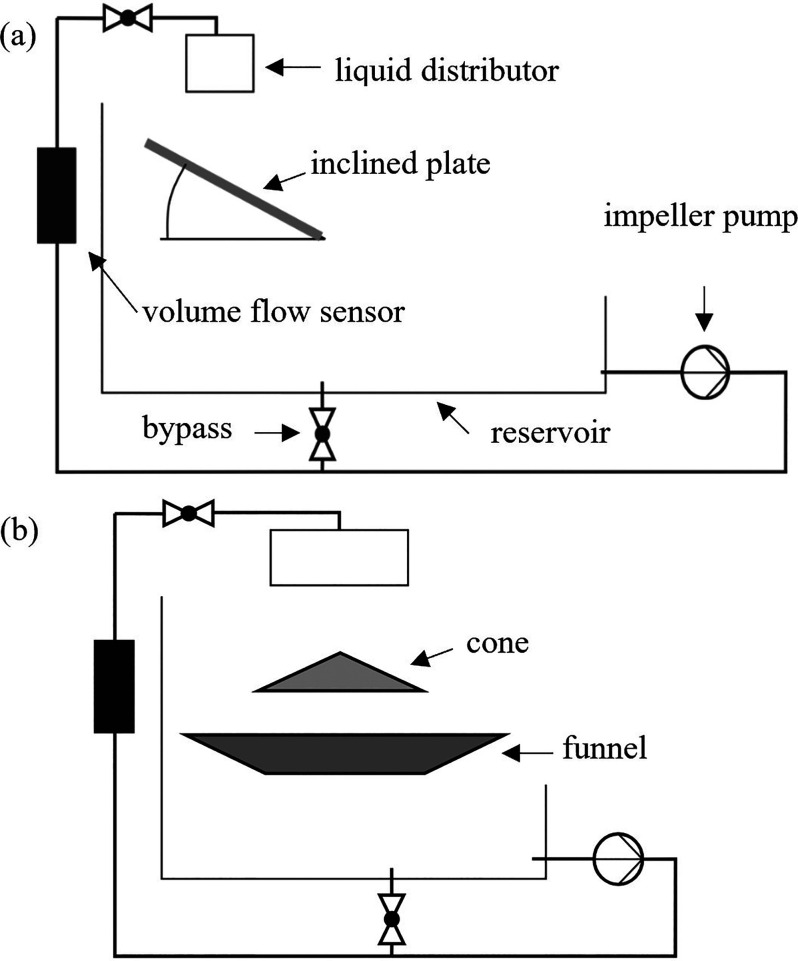
Schematic layout of the experimental setup with the inclined plate^15^ (a) and with CAF (b) (Reproduced from S.A. Bernemann et al., *Chem. Eng. Sci.,* **2024**, 300, 120639, under the terms of the CC BY-NC-ND 4.0 license).

**Figure 3:**
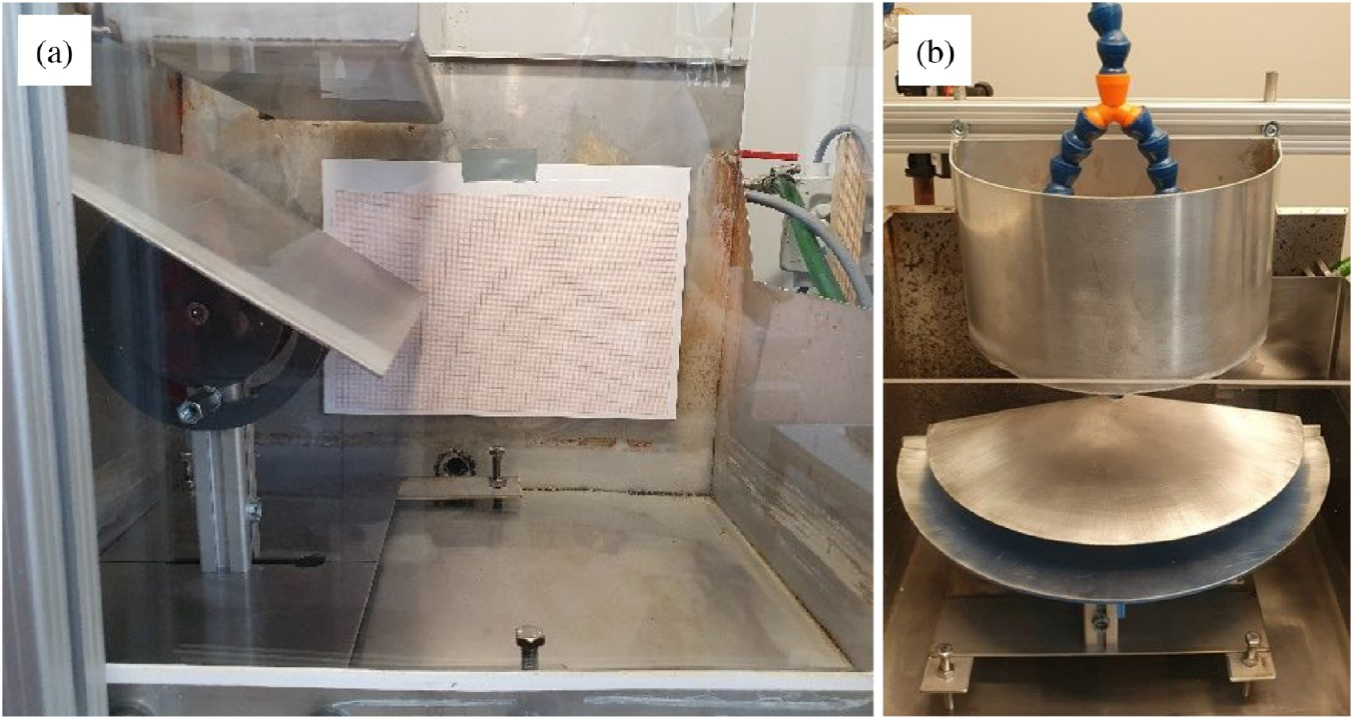
View through the window of the experimental setup onto the plate (a)^15^ and adapted layout with CAF inside the reservoir (b) (Reproduced from S.A. Bernemann et al., *Chem. Eng. Sci.,* **2024**, 300, 120639, under the terms of the CC BY-NC-ND 4.0 license).

In the experiments, two geometries of the CAF designed for a nominal diameter of DN400, one with 30° (Figure 4) and another with 10° (Figure 5) surface angles to the horizon, were examined. The second geometry with a 10° angle was suggested as a step toward performance optimization; the details are described below in Section 3.1.2. Due to the space limitations of the reservoir, only halves of the CAFs were used (cf. Figure 3b).

**Figure 4:**
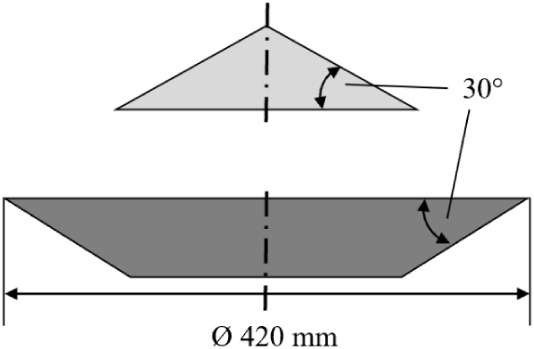
30° CAF geometry for a nominal diameter of DN400.

**Figure 5:**
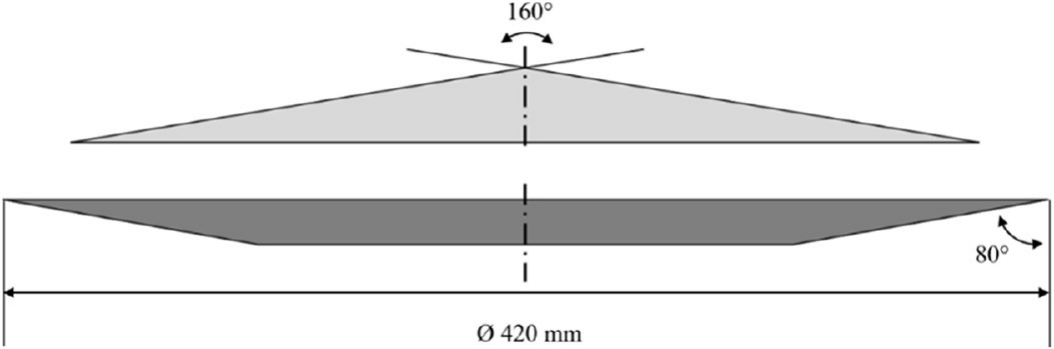
10° CAF geometry for a nominal diameter of DN400.

To fabricate the structures precisely reproducing the planned geometries of the CAF, a base was 3D-printed out of plastic. A sheet of aluminum was glued onto the base to achieve the desired surface finish. For the flat plate, a linear liquid distributor expanded across the entire width of the plate was used, with ten 8 mm holes spaced 25 mm apart along the center line in the bottom (cf. Figure 6a). For the CAF geometries, a circular distributor with six 8 mm holes distanced 30° from each other on a circle with a 50 mm radius was applied (cf. Figure 6b).

**Figure 6:**
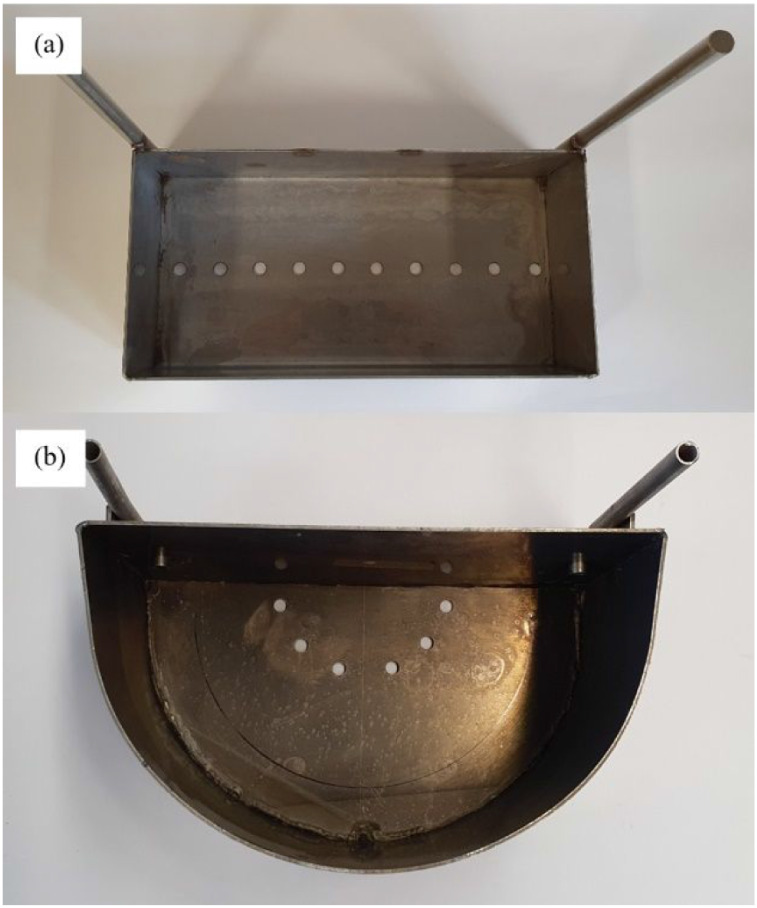
Linear liquid distributor (a) and circular liquid distributor (b).

#### 2.2.2 Measuring Method

Measurements were performed of the vertical (*y* in Figure 7) and horizontal (*x* in Figure 7) distances of the liquid from the edge of the inclined plate. This was accomplished by taking photographs of the fluid stream from the side of the plate through the window, as shown in Figure 7, with a Canon EOS 90D and the settings listed in Table 1. The position of the fluid was subsequently measured with the software Graph Grabber v2.0.2 from Quintessa Limited (cf. Bernemann et al.^15^). In the photograph, the outline of the liquid jet is marked at a height of *y* = −100 mm with the points *x*_1_ and *x*_2_ (cf. Figure 7). The center of the jet is then determined by the following equation:

1 $$x\left(y=-100\mathrm{mm}\right)=\frac{x_{1}+x_{2}}{2}$$

**Figure 7:**
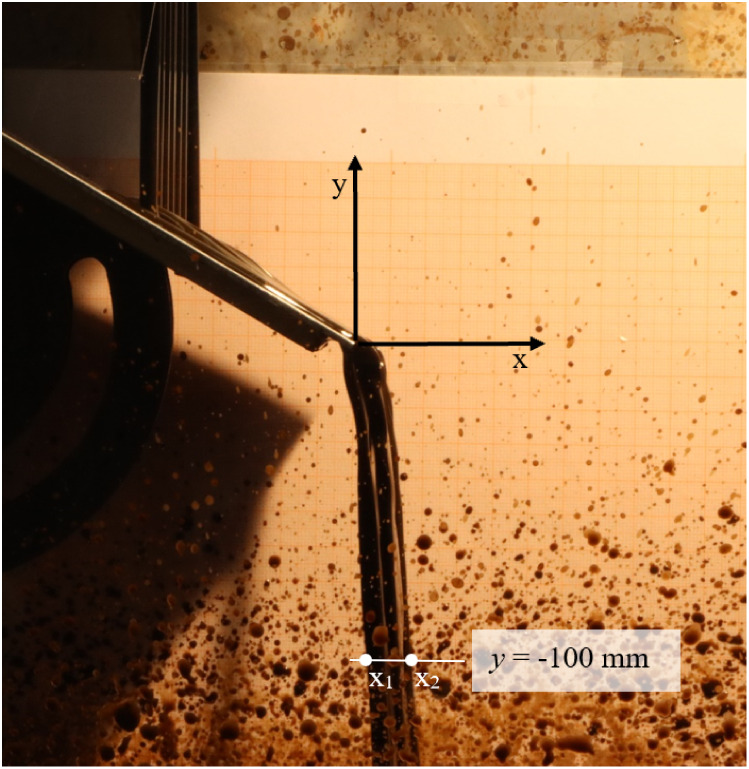
Measurement photo with the reference frame and measurement points.

**Table 1:** Camera Settings for Experimental Measurements

Setting	Value
Resolution	6960 × 4640
Aperture	F22
Exposure time	1/50 s
ISO	2000

To obtain reliable results by using this method, the experimental measurements were repeated several times for each flow rate. To account for flow fluctuations, each measurement was based on three photographs made for a selected flow rate. The mean values and standard deviations of the results were then used for the analysis.

### 2.3 CFD Modeling

Three-dimensional CFD simulations were performed by using Ansys Fluent 2021 R2. The CFD model describes a two-phase system, consisting of gas and liquid, under the assumptions of incompressible, isothermal, and laminar flow conditions.

#### 2.3.1 Governing Equations

The flow was governed by the differential equations for the conservation of mass and momentum, which can be written as follows:

2 $$\frac{\partial \rho }{\partial t}+\nabla \cdot \left(\rho u\right)=0$$

3 $$\frac{\partial }{\partial t}\left(\rho u\right)+\nabla \cdot \left(\rho uu\right)=\nabla S+\rho g-\nabla p+f_{\sigma }$$

In these equations, ρ denotes the fluid density, *t* represents time, ***u*** is the velocity vector, ***S*** is the viscous stress tensor, ***g*** is gravity, *p* is pressure. The term ***f***_σ_ represents a volume source that describes surface tension forces according to Brackbill et al. (1992).^16^ The non-Newtonian rheological behavior of the liquid waste was characterized using the following power-law model.^17^

4 $$\eta =K\cdot {\mathrm{\gamma ̇}}^{n-1}$$

Here, η denotes the dynamic viscosity, *K* is the consistency index, γ̇ represents the shear rate, and *n* is the flow behavior index. The volume of fluid (VoF) method^18^ was employed to simulate the multiphase flow and to capture the interface between the gas and liquid phases. This approach introduces an additional scalar, the volume fraction α_L_, which quantifies the fraction of liquid in a certain volume. A value of *α*_L_ = 1 indicates a cell entirely filled with liquid, whereas α_L_ = 0 corresponds to a cell filled with gas. Volume fractions of 0 < α_L_ < 1 represent cells at the gas–liquid interface, which results in a smeared interface over a certain volume. The transport of the volume fraction is governed by the following equation:

5 $$\frac{\partial \alpha_{L}}{\partial t}+u\cdot \nabla \alpha_{L}=0$$

The volume forces ***f***_σ_ (cf. eq 3) are defined according to the continuum surface force (CSF) model:^16^

6 $$f_{\sigma }=\sigma \kappa n$$

Here, σ is the surface tension, κ is the curvature, and ***n*** is the surface normal which is given by

7 $$n=\nabla \alpha_{L}$$

The curvature is calculated with the following equation:

8 $$\kappa =-\nabla \cdot \frac{n}{\left|n\right|}$$

#### 2.3.2 Computational Domains and Boundary Conditions

For the simulation studies, the model developed by Bernemann et al.^15^ was used. The majority of the simulations were performed in two computational domains: one representing the space between the liquid distributor and the flat steel plate and the other capturing the region around the lower edge of the plate. These domains correspond to the experimental setup described in Section 2.2.1; they are shown in Figure 8. The width of the computational domains along the *z*-axis was set to 25 mm, to be in line with the spacing of the holes in the distributor.

**Figure 8:**
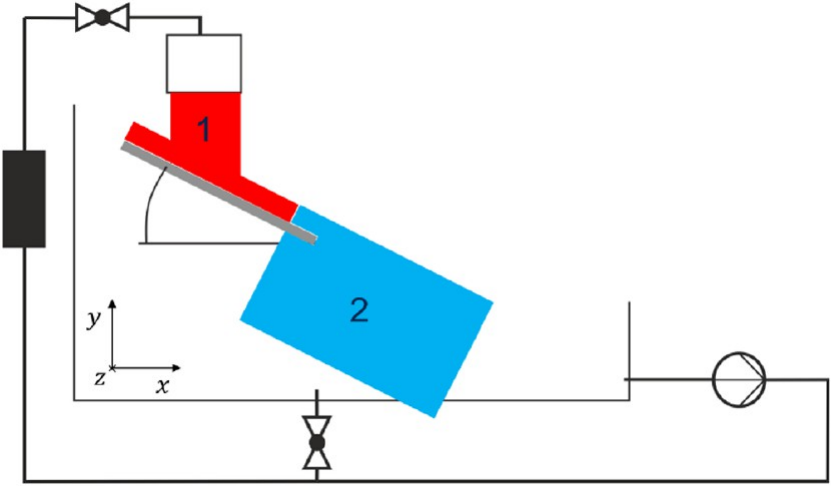
Position of computational domains 1 and 2 within the experimental setup^15^ (Reproduced from S.A. Bernemann et al., *Chem. Eng. Sci.,* **2024**, 300, 120639, under the terms of the CC BY-NC-ND 4.0 license).

The simulated domains were connected at their common boundary, while the volume fraction and velocity profiles obtained in the simulation in domain 1 were used as inlet conditions for the simulation in domain 2. This method is applicable only to simulations with a steady-state velocity profile at the connecting boundary. This limitation arises because ANSYS Fluent does not support the data transfer of the time-resolved profiles. However, some of our simulations did not reach the necessary steady state with the stationary profiles. This made it necessary to combine computational domains 1 and 2 into a single large domain as shown in Figure 10.

The boundary conditions in accordance with the numbered regions are summarized in Table 2. At the top (boundary 1), there is a flow inlet with defined profiles for the velocity and volume fraction corresponding to the outlet of the liquid distributor, as shown in Figure 9.

**Figure 9:**
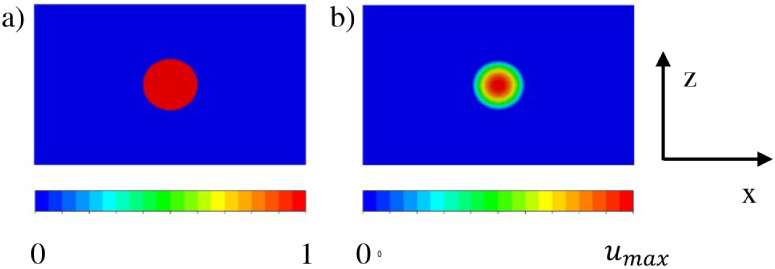
Volume fraction (a) and velocity profile (b) at the inlet boundary of domain 1 ^15^ (Reproduced from S.A. Bernemann et al., *Chem. Eng. Sci.,* **2024**, 300, 120639, under the terms of the CC BY-NC-ND 4.0 license).

**Table 2:** Boundary Conditions Used in the CFD Model (Numbers Corresponding to Figure 10)

location	boundary	mathematical condition
1	inlet	***u***, α_L_ as predefined profiles
2	outlet	***p*** = 1 bar, ∇α_L_ = 0
3	wall	***u*** = 0, α_L_ = ***f*** (θ)
4	symmetry	***u*** · ***n***_wall_ = 0, ∇α_L_ = 0

The surfaces marked with 2, located at the top, bottom, front, and back sides, are defined as outlets with a constant pressure of 1 bar. To reduce the width of the computational domain, symmetry boundary conditions are applied to the side surfaces marked as 4.

The locations denoted with 3 represent the surface of the steel plate; therefore, a no-slip wall boundary is applied. In these locations, a variable contact angle is utilized. In the cells adjacent to the no-slip wall boundary, the unit vector ***n*** is defined as follows:

9 $$n=n_{\mathrm{wall}}\cdot \cos\Theta +t_{\mathrm{wall}}\cdot \sin\Theta$$

with ***n***_wall_ and ***t***_wall_ being the unit vectors normal and tangential to the wall. The contact angle Θ depends on the velocity of the contact line ***u***_cl_. At negative velocities, at which the contact line moves in the direction of the liquid, the receding contact angle Θ_R_ is used. When the velocity of the contact line is zero, the static contact angle Θ_S_ is applied. When the liquid spreads on the surface resulting in positive velocities of the contact line, the advancing contact angle Θ_A_ is used.

#### 2.3.3 Meshing

The three-dimensional computational domains described in Section 2.3.2 are discretized into small hexahedral cells using meshes generated with the software ICEM 2020 R1 from Ansys. A high mesh resolution is essential for achieving accurate simulation results; however, it also significantly increases the computational demands. To ensure mesh independence of the simulations, a mesh must be refined until further refinement does not substantially influence the results. Bernemann et al.^15^ reported that this condition was fulfilled with a mesh containing 2.8 million cells for domain 1 and 17 million cells for domain 2. For the combined computational domain (Figure 10), the cell size of computational domain 2 was applied everywhere in order to capture the characteristics of the flow with high accuracy. As a result, the total mesh size increased to 25 million cells.

**Figure 10:**
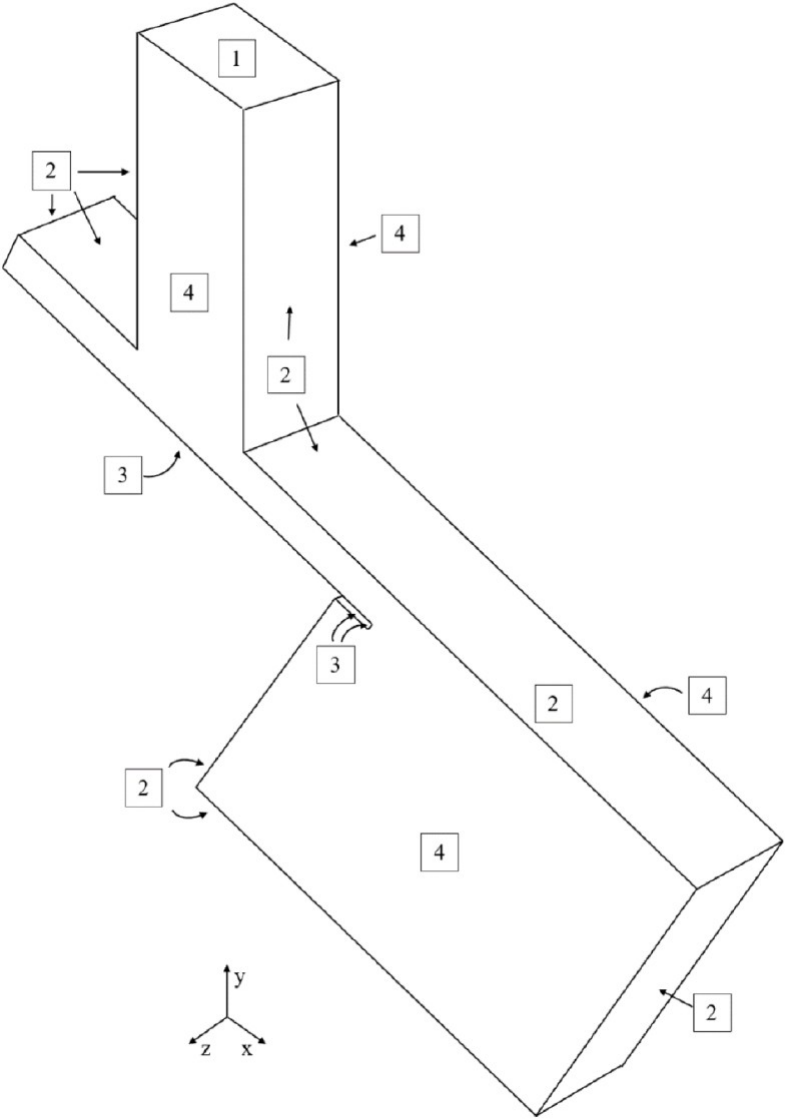
Combined computational domain for the whole geometry with boundary conditions listed in Table 2.

#### 2.3.4 Solver Settings

For spatial discretization of the governing equations, the second-order upwind scheme was used. Pressure–velocity coupling was realized through the application of the Pressure-Implicit with Splitting of Operators (PISO) scheme. To improve the accuracy of the interface localization, the piecewise-linear interface calculation (PLIC) method was applied. Convergence of the simulations was evaluated based on multiple criteria. First, relative deviations between consecutive values for all process variables were required to fall below a threshold of 0.001. In addition, key computed parameters, such as the liquid holdup and wetted area, had to reach constant values.

## 3 Results and Discussion

The flow of liquid agricultural waste was analyzed using both experimental investigations and CFD simulations. Initially, the flow on the 30° cone was investigated in the experimental setup regarding the complete wetting of the CAF while geometrical improvements of the CAF structure were identified. These improvements were realized and examined experimentally. Furthermore, using the simpler flat plate structure, residue accumulation was studied. The CFD model was utilized to examine the influence of various physical properties of agricultural waste, including contact angle, surface tension, and viscosity, on flow behavior.

### 3.1 Experimental Studies

#### 3.1.1 30° CAF Configuration

To assess the wetting of the CAF, first, the cone with a 30° angle was positioned beneath the liquid distributor with a circular hole pattern (cf. Figure 2b). The cone was wetted with the liquid agricultural waste with volumetric flow rates of 3, 6, 9, 12, 15, and 18 L/min. The lowest flow rate value was dictated by the detection limit of the volumetric flow sensor, while the highest value was determined by the maximum possible fluid level in the distributor and the resulting gravity-driven outflow. The experiment showed that even at the lowest flow rate of 3 L/min, the cone was completely wetted, as illustrated in Figure 11. However, it was observed that under these conditions, the liquid dropped vertically from the edge of the cone. This would hinder the wetting of the funnel underneath. For this reason, no experiments with a complete 30° CAF were conducted.

**Figure 11:**
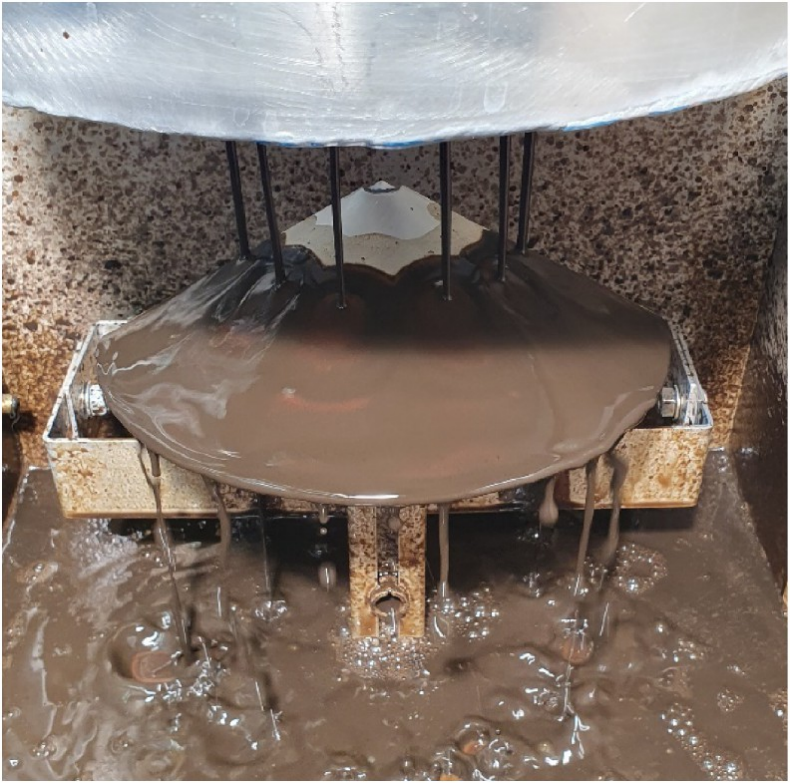
Liquid agricultural waste on the 30° cone at a volumetric flow rate of 3 L/min.

With the studied 30° angle configuration, surface wetting of the cone is perfect, but the primary goal to provide reasonable wetting of the complete CAF configuration cannot be achieved. Therefore, in the following step, an attempt was made to adjust the CAF geometry in such a way that a closed liquid film could be established on the funnel surface, too. In principle, increasing the widths of the cone and funnel leads to a larger overlap between these two parts and hence enhances the wetting. With a 30° surface inclination, however, the widening of the CAF would lead to an increased height. This can be avoided by inclination reduction, and we chose the value of 10°, which is the lowest inclination used in our previous work.^15^ The evaluation of this change in the CAF geometry is highlighted in the following section.

#### 3.1.2 New Cone and Funnel System

Based on the findings described above in Section 3.1.1, a new CAF shown in Figure 5 was suggested. In the new version, both cone and funnel widths were increased, yielding a higher overlap between the two CAF parts. The maximum width of the column internals was determined for the column diameter of DN400 and the maximum possible gas velocity resulting from the free cross-sectional area and the desired volumetric gas flow in the application. The chosen column diameter of DN400 represents the dimension appropriate for actual use in biogas plants, thus eliminating the scale-up problem. The modification increases the overall surface area of the CAF, which, assuming optimal wetting, enhances the gas–liquid interfacial area. Furthermore, the inclination angle was reduced to 10°, resulting in a lower height of the internals and hence enabling a greater number of cones and funnels to be stacked within a given column height. This results in a 30% higher surface area for each new CAF with a 10° angle. Since the number of 10° CAFs placed within a given column segment is higher, a surface area over three and a half times bigger than that of the CAF with a 30° angle can be achieved. The new CAF with a 10° angle was tested at volumetric flow rates ranging from 3 to 18 L/min, corresponding to liquid loads of 2.6 to 15.6 m^3^/m^2^ h. At the lowest flow rate (3 L/min) and without prewetting, complete wetting of the CAF could not be achieved. However, full wetting was observed when the surface was prewetted using the same agricultural waste at a higher flow rate, as illustrated in Figure 12.

**Figure 12:**
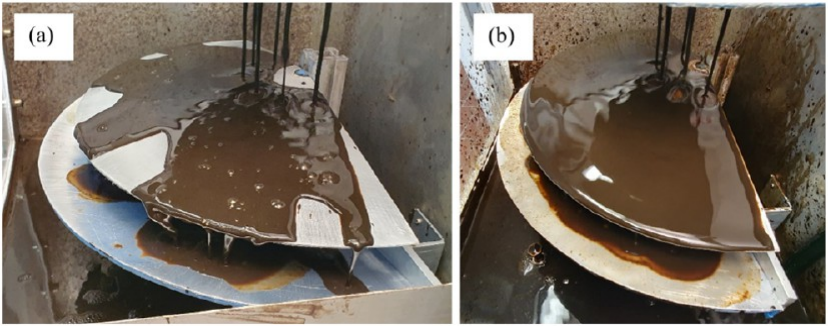
Partial wetting on a dry surface (a) and full wetting on a prewetted surface (b) on the 10° cone at a volumetric flow rate of 3 L/min.

A problem was encountered with the construction of the plastic support structure on the underside of the cones and funnel. This support was extended up to the edge of the metal sheet, as can be seen in Figure 13a. This caused backflow on the underside of the CAF, reducing the wetting of the lower structure, as shown in Figure 13b.

**Figure 13:**
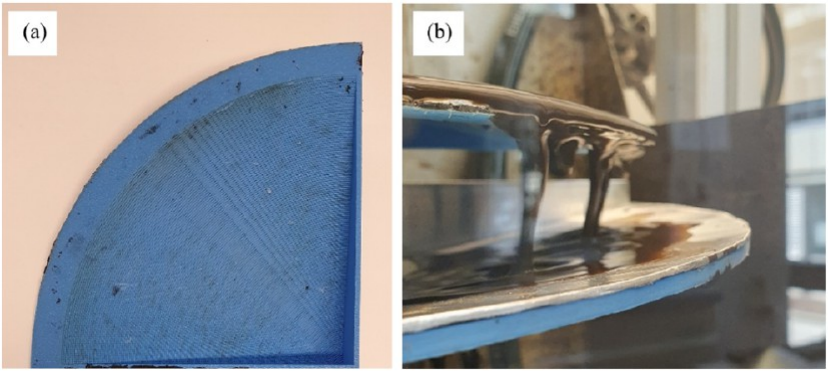
Underside of the 10° cone with plastic support structure (blue) up to the edge (a) and the corresponding backflow on the underside of the cone (b).

To solve this problem, 20 mm of the support structure adjacent to the edge was removed (Figure 14a). The resulting sharp tear-off edge ensured that the liquid mostly detached from the edge of the cone without running back to the underside (Figure 14b).

**Figure 14:**
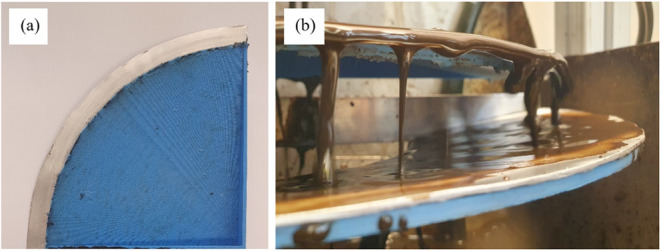
Underside of the cone with the plastic support structure (blue) removed at the edge (a) and corresponding reduced backflow on the underside of the cone (b).

Another problem was that, at higher volumetric flow rates, the momentum of the liquid flowing from the cone to the funnel caused the fluid to spill over the outer edge of the funnel, as shown in Figure 15a. This overflow would result in an undesired near-wall flow in a column. To mitigate this effect, a vertical wall was attached to the outer edge of the funnel (Figure 15b and Figure 16). This wall was made of a 50 mm high aluminum strip mounted to a quarter of the funnel, as shown in Figure 16. In addition to preventing overflow, this wall may function as a wall wiper when it is used in a stripping column.

**Figure 15:**
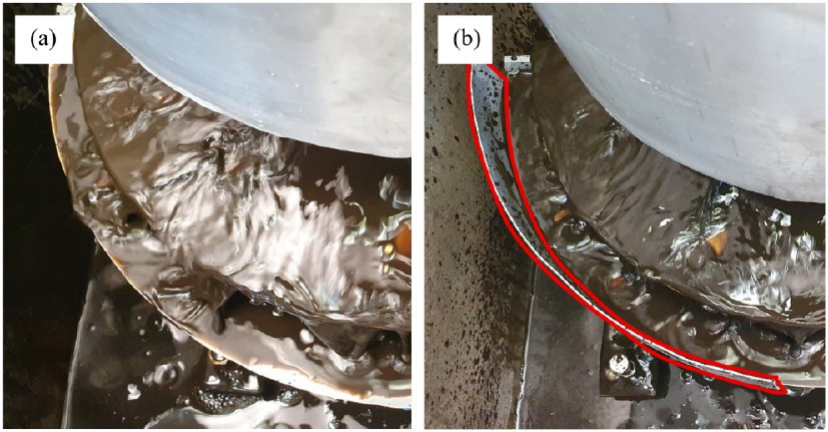
Spilling of the liquid over the outer edge of the funnel (a) and its prevention through a vertical wall at the edge highlighted in red (b) at a volumetric flow rate of 18 L/min.

**Figure 16:**
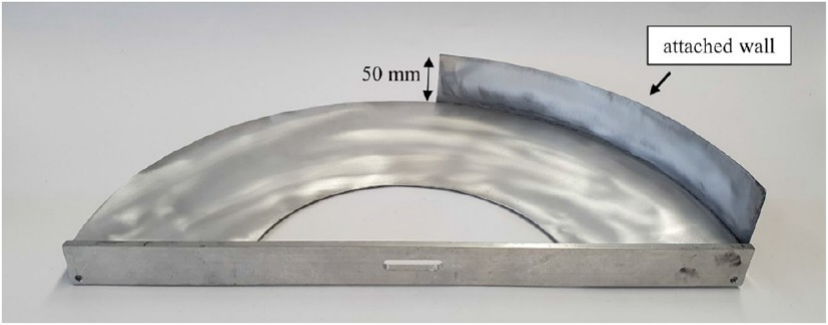
Funnel segment of the CAF structure with an attached partial vertical wall at the outer edge.

The revised structure, incorporating these adjustments, was tested and demonstrated an improved performance, achieving full surface wetting. However, due to the limitations of the laboratory setup, the effects of countercurrent gas flow and the efficiency of the new structure as column internals could not be assessed. Further evaluation in a demonstration-scale system, therefore, is required.

#### 3.1.3 Laboratory Plate System

A primary objective in developing the new column internals was to reduce the susceptibility to deposits from agricultural waste. Such deposits are formed over extended operation periods of stripping columns, while their buildup is amplified by hot stripping gases. Since an industrially relevant operation with countercurrent hot gas can hardly be investigated in the laboratory setup over long periods, an alternative experimental approach was used. Five consecutive experimental runs were conducted, with the residues subjected to drying between the subsequent runs. This way helps to promote the formation of residue accumulation on the inclined plate, similar to the action of hot gas in real operations. The suggested procedure enables the analysis of the formed residue and the assessment of its impact on the flow behavior of the agricultural waste. The residue buildup after each of the five runs is shown in Figure 17. A progressive darkening of the residue is observed, indicating an increase in the thickness over successive experiments.

**Figure 17:**
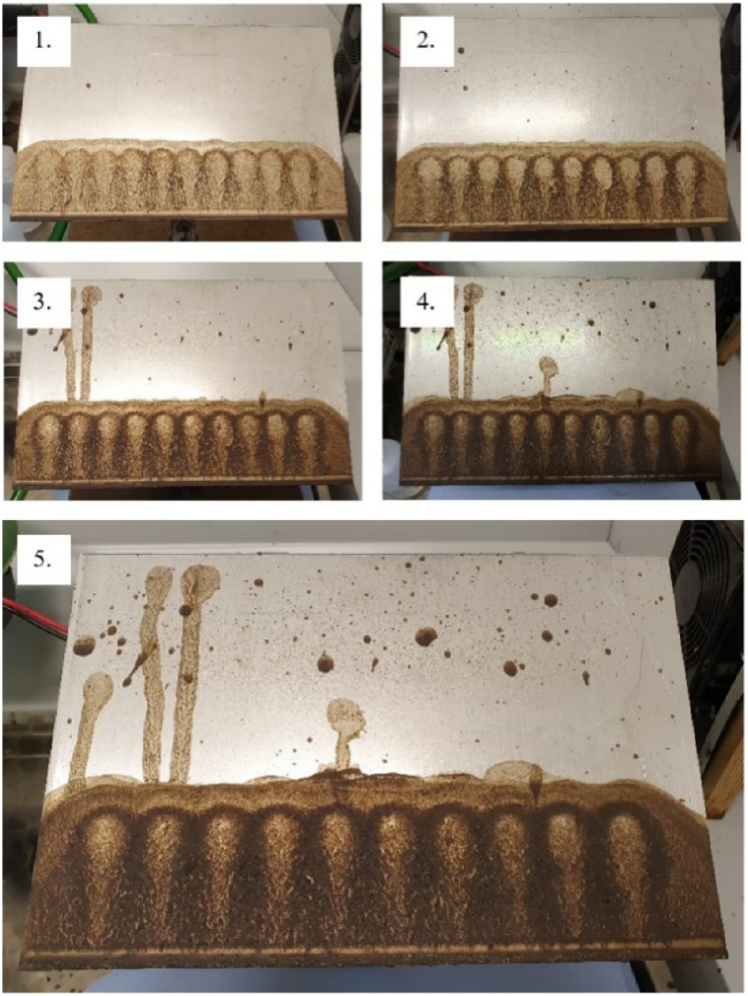
Progressively darker color of dried residue on the inclined plate after 1 to 5 repetitions of the experiment indicates increasing residue thickness.

However, the new column internals (cf. Figures 4 and 5) are designed with sufficient open space around the edges, reducing the risk of column clogging even with extensive residue accumulation and thick deposit layers. With conventional random packings, as previously used at ENVIMAC Engineering GmbH, these deposits lead to total choking of the flow path (Figure 18), leading to column clogging. With the new internals, such clogging would be prevented, and the operation would not be jeopardized by the residue, ensuring stable and reliable performance in the stripping column, even over long periods.

**Figure 18:**
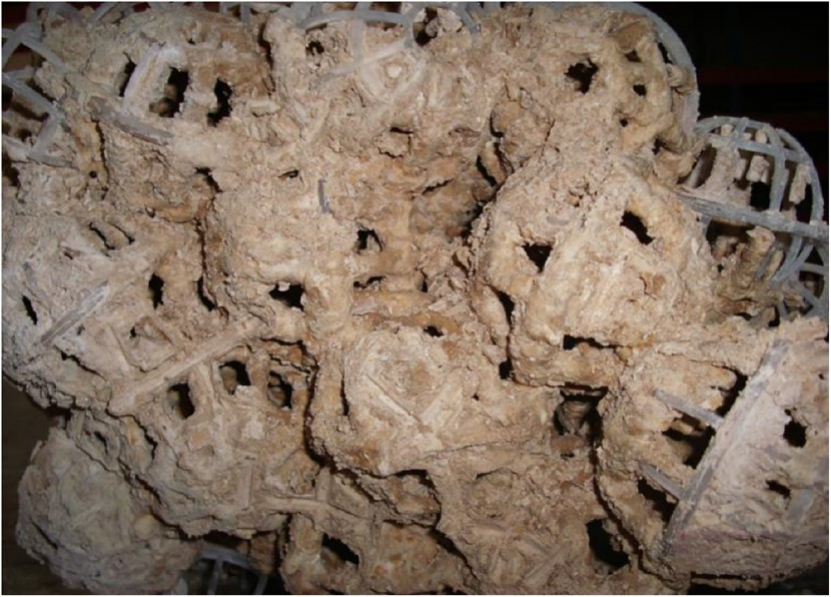
Solid residue on conventional random packings leading to clogging of the column during ammonia stripping from agricultural waste. Photo by ENVIMAC Engineering GmbH.

The effect of residue buildup on the separation of the liquid agricultural waste from the lower edge of the inclined plate was also examined. The results for all five repeated experiments are depicted in Figure 19. No significant influence of residue accumulation on the distance *x* or the overall flow behavior was observed. These findings demonstrate the high robustness of the design against residue buildup, which was a key objective for its development.

**Figure 19:**
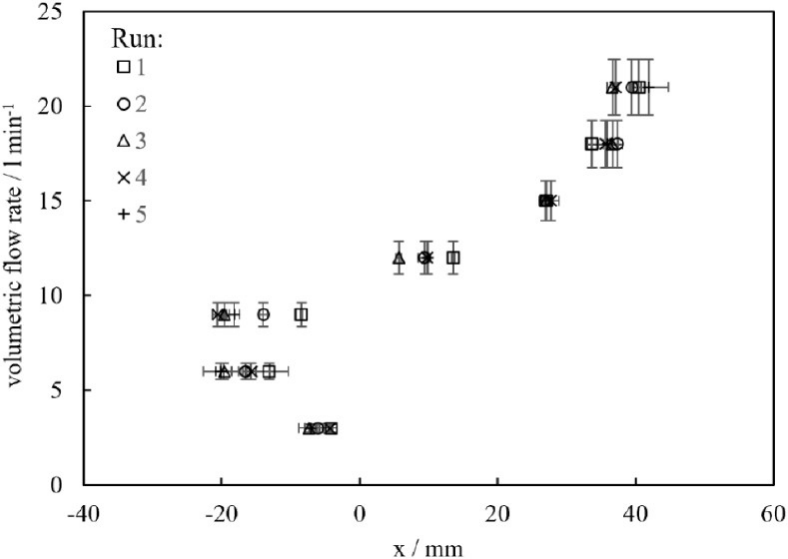
Distance *x* over the volumetric flow rate after 1 to 5 experimental runs with increasing residue buildup.

### 3.2 Computational Studies

As described above, experimental investigations helped to improve the CAF geometry. They were complemented by computational studies aimed at finding beneficial operating conditions. The focus was on the influence of the volumetric flow rate and the physical properties of the agricultural waste on the flow behavior.

#### 3.2.1 Contact Angle

Previous studies^15^ revealed that the performed CFD simulations did not accurately replicate the flow behavior observed in experiments for a plate inclination angle of 50° and a volumetric flow rate of 3 L/min. While the experiment showed complete wetting of the plate, the simulation resulted in the formation of a rivulet. It was supposed that the prewetting of the plate could lead to the closed film flow in the experiment. To assess this hypothesis, in the present work, a simulation was conducted with an initially increased flow rate to induce prewetting. A volumetric flow rate of 9 L/min was applied for the first 2 s of simulated time, followed by a gradual reduction to 3 L/min over the next 2 s. As shown in Figure 20 (left), the higher initial volumetric flow rate resulted in a closed film at a simulated time instant of 2 s. However, after reducing the flow rate at 6 s, a film reduction can be observed around the jet impingement point (Figure 20, middle). Over time, this reduction propagates downward, leading to the formation of a rivulet as shown in Figure 20 (right) for a flow duration of 10 s. This rivulet formation is similar to that obtained in the simulation without prewetting.

**Figure 20:**
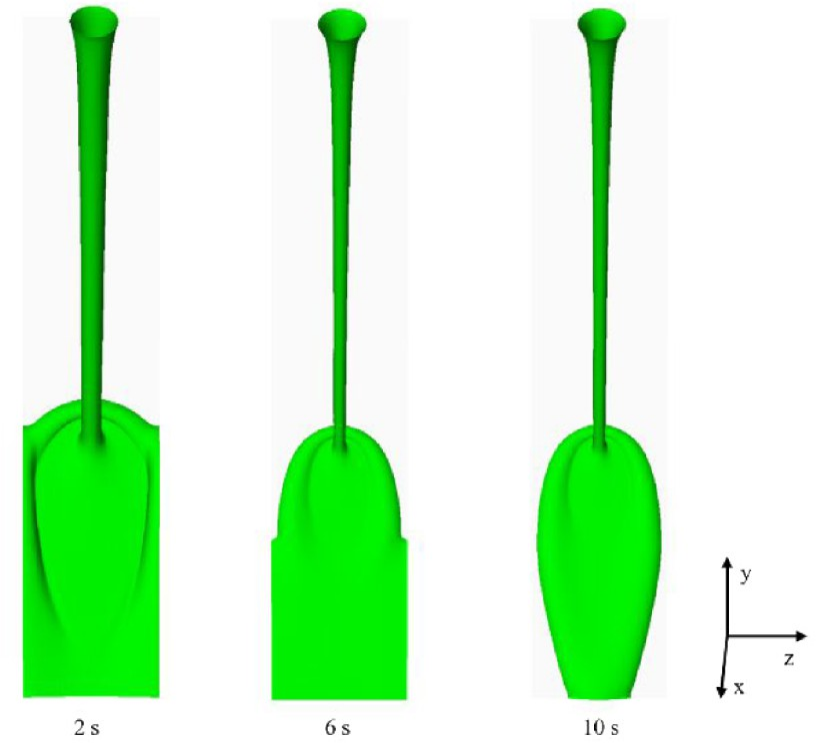
Formation of a rivulet at a plate inclination angle of 50° with a reduction of the volumetric flow rate from 9 L/min to 3 L/min at 2, 6, and 10 s of simulated time. The gas–liquid interface is marked green.

The experiments conducted by Bernemann et al.^15^ showed that areas where the fluid receded never fully dried. This observation indicates good wetting behavior and low contact angles. To mirror this behavior, the simulation with the reduced flow rate was repeated with the receding contact angle set to 0°. This adjustment led to the formation of a stable closed film, as shown in Figure 21, which more closely conforms to the experimental results. These findings suggest that the receding contact angle of 35° measured by Bernemann et al.^15^ and used as a parameter in the simulations was inaccurate. The inaccuracy was most likely caused by the absence of solid content in the measured agricultural waste which was necessitated by the measurement method.^15^

**Figure 21:**
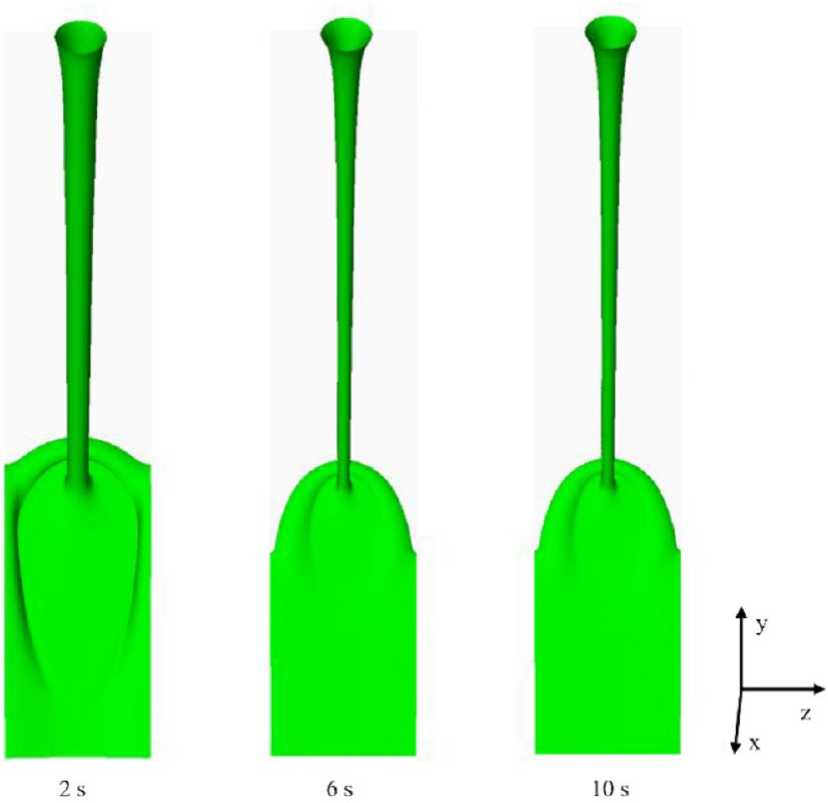
Formation of a film with a plate inclination angle of 50° with a reduction of the volumetric flow rate from 9 L/min to 3 L/min at 2, 6, and 10 s of simulated time and a receding contact angle equal to 0°. The gas–liquid interface is marked green.

Furthermore, the influence of variations in the static and advancing contact angles on flow behavior was examined for the flow rates of 3, 9, 15, and 21 L/min. An inclination angle of 30° was selected, corresponding to the proposed cone geometry. This configuration was also used for all subsequent simulation studies. In this study, the values of the static and advancing contact angles were varied as shown in Table 3, while the receding contact angle remained fixed at 0°.

**Table 3:** Variation of the Static and Advancing Contact Angle

Variation	–20%	–10%	0%	+10%	+20%
θ_A_	52°	58.5°	65°	71.5°	78°
θ_S_	48°	54°	60°	66°	72°

The simulation results without modifications to the static and advancing contact angles are shown in Figure 22 (left) in comparison with the simulation and experimental data for the inclination angle of 30° taken from Bernemann et al.^15^ This comparison was carried out to assess the effect of setting the receding contact angle to 0°. The simulations with a receding contact angle of 0° exhibited lower deviation from the experimental data for all flow rates studied, supporting this assumption.

**Figure 22:**
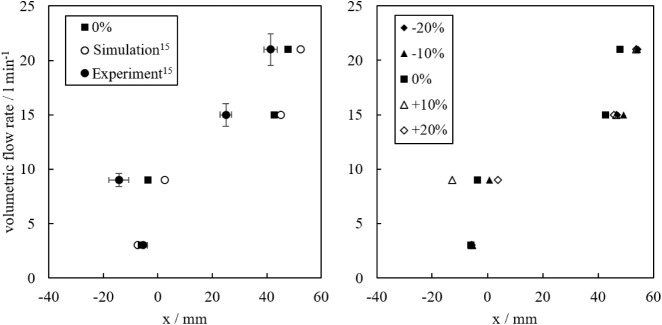
Simulation results with a 0° receding contact angle and without variation of static and advancing contact angles in comparison to the simulation and experimental results from Bernemann et al.^15^ (left) and simulation results for varied static and advancing contact angles according to Table 3 (right) (Reproduced from S.A. Bernemann et al., *Chem. Eng. Sci.,* **2024**, 300, 120639, under the terms of the CC BY-NC-ND 4.0 license).

Figure 22 (right) displays the simulation results for variations in the static and advancing contact angles according to Table 3. In the investigated range of volumetric flow rates, no clear trend in the contact angle’s impact on the distance *x* can be recognized. This means that changing the surface material, and hence the contact angle, cannot be used to optimize flow behavior. Furthermore, the influence of the contact angle on the distance *x* is low compared with the impact of the flow rate. We also found no impact of the static or advancing contact angle on the wetting behavior. Consequently, variations in the wetting behavior of the fluid, such as those naturally occurring in agricultural waste, would hardly influence the overall process.

#### 3.2.2 Surface Tension

The flow topology of agricultural waste can also be influenced by surface tension. The latter can be modified using surface-active additives, such as antifoam agents. The impact of surface tension was investigated in another simulation study. The values of the surface tension σ were set as deviations from the basic value measured by Bernemann et al.;^15^ they are listed in Table 4. The corresponding values of *x* for volumetric flow rates changing from 3 to 21 L/min are presented in Figure 23 in comparison with the simulation results of Bernemann et al.^15^ obtained with the basic surface tension value.

**Table 4:** Variation of the Surface Tension

Variation	–20%	–10%	0%	+10%	+20%
σ [mN/m]	38.4	43.2	48 ^15^	52.8	57.6

**Figure 23:**
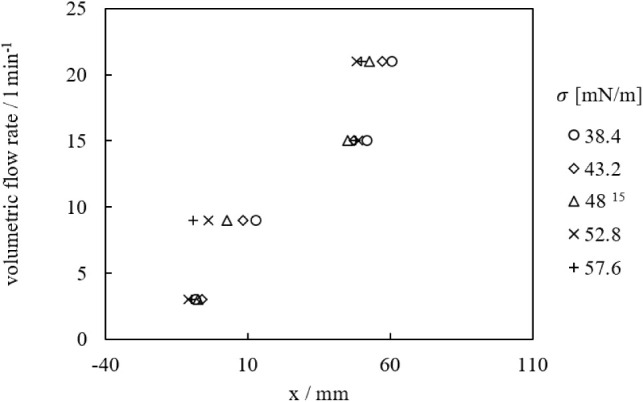
Simulated flow rates for varied surface tension values in comparison with the simulation results of Bernemann et al.^15^

The most significant impact on the distance *x* is again observed at a flow rate of 9 L/min, which is in line with the findings from the contact angle study. At this flow rate, a lower surface tension value corresponds to higher *x* values, and vice versa. The flow rate influence on the distance *x* is clearly more significant than the influence of surface tension.

A notable impact of the surface tension at the flow rate of 9 L/min is evident in the graph showing the dependence of the gas–liquid interfacial area on the volumetric flow rate (Figure 24). A substantial gap is visible between the values obtained for 43.2 and 48 mN/m, with a lower surface tension leading to larger interfacial areas. However, no clear trend could be established for other flow rates.

**Figure 24:**
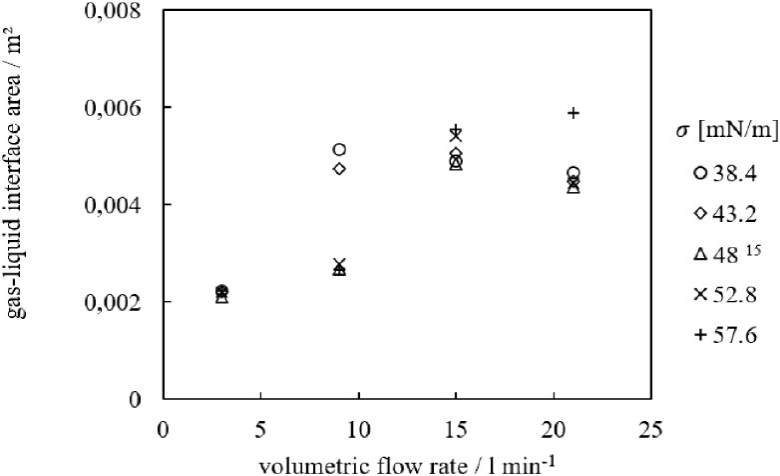
Simulated gas–liquid interface area in domain 2 over the volumetric flow rate for varied surface tensions.

The difference in interfacial area size arises due to the formation of a thin liquid film in the free-fall regime, which subsequently splits into three jets at surface tensions of 43.2 mN/m and lower. In contrast, at surface tensions of 48 mN/m and higher, the liquid directly separates from the plate edge in the form of two jets, which results in a lower interfacial area. These flow specifics are illustrated in Figure 25 for surface tension values of 43.2 and 52.8 mN/m.

**Figure 25:**
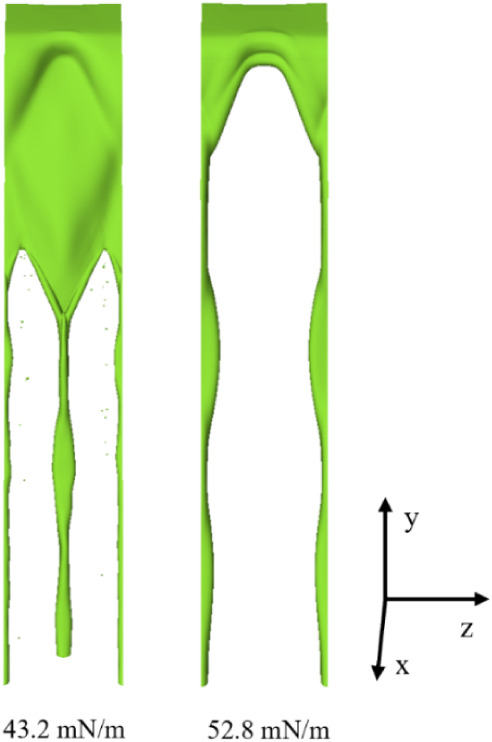
Gas–liquid interface (green) at 9 L/min and surface tension values of 43.2 and 52.8 mN/m.

However, no general trend can be recognized for the influence of surface tension on the gas–liquid interface area. Therefore, additives reducing the surface tension of the liquid agricultural waste, like antifoam additives, cannot be applied to increase the interface area. It is also worth noting that real flow behavior within an apparatus is likely influenced by the countercurrent gas flow; this influence was considered in neither the simulation nor the experimental study.

#### 3.2.3 Viscosity

Viscosity measurements performed before and after a series of experiments showed a significant reduction in viscosity values. To evaluate the influence of this change on the flow behavior independently of other physical properties, a further simulation study was carried out. The simulations were conducted by using both the measured viscosities (before and after the measurements) and three additional theoretical viscosity values. The shear rate dependence of these viscosities is presented in Figure 26, where number 1 represents the highest and number 5 represents the lowest theoretical viscosity.

**Figure 26:**
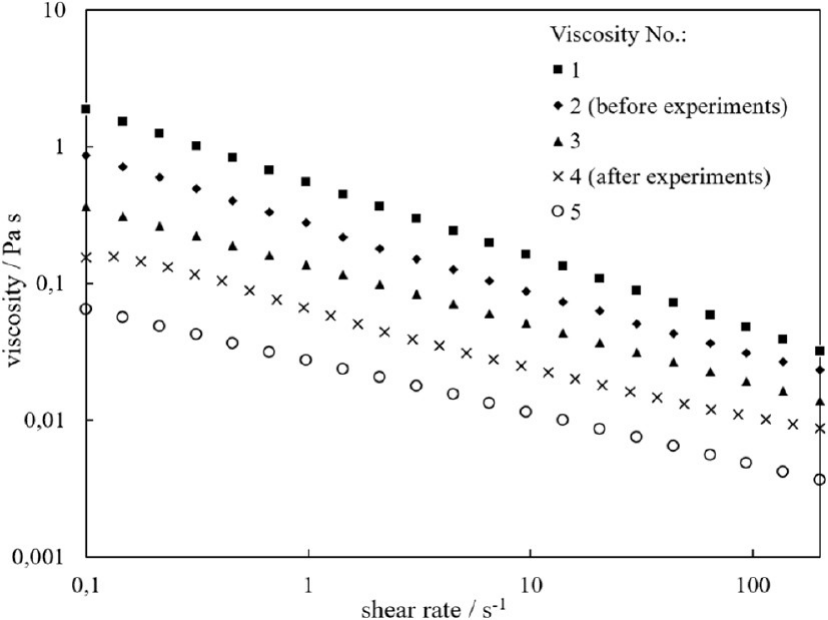
Shear rate dependency of the viscosities from Table 5 used in the simulation study.

The consistency index and the flow behavior index for the power-law model are given in Table 5 for all five shear-rate-dependent viscosities. The simulations with volumetric flow rates of 15 and 21 L/min using viscosities No. 4 and No. 5 did not reach fully stationary profiles in domain 1 due to the formation of transient waves in the fluid film. For this reason, the combined computational domain depicted in Figure 10 was applied, as explained in Section 2.3.2.

**Table 5:** Power-Law-Model Parameters for Variation of the Shear Rate-Dependent Viscosities

Viscosity No.	1	2 (before experiments)	3	4 (after experiments)	5
*K*	0,5432	0,2716	0,1358	0,0656	0,02716
*n*	0,4653	0,517	0,5687	0,591	0,6204

The simulation results presented in Figure 27 indicate no significant effect of viscosity for a low volumetric flow rate of 3 L/min. However, at higher flow rates, lower viscosities bring about an increase in distance *x*. This trend is particularly pronounced at a flow rate of 9 L/min, where the highest viscosity (No. 1) results in *x* = −16.2 mm, while the lowest viscosity (No. 5) leads to *x* = 50.9 mm.

**Figure 27:**
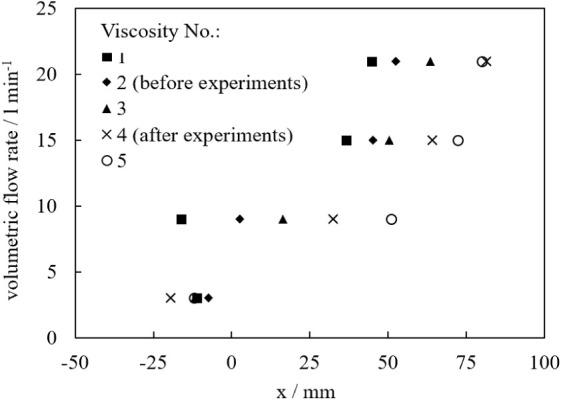
Simulated volumetric flow rates for different viscosities shown in Table 5.

In the CAF structure, a higher *x* value is preferred as this results in an increased wetted area of the subsequent structure (cone or funnel) below. Therefore, a reasonable preprocessing of the ammonia removal process toward the reduction of the liquid agricultural waste viscosity, thereby maximizing the wetted area of the column internals, appears suitable.

## 4 Conclusions and Outlook

This study was focused on preventing clogging in stripping-based ammonia removal from liquid agricultural waste. Innovative stripping column internals designed for this separation process were investigated using both experimental and computational fluid dynamics (CFD) methods. An initial design comprising a cone and a funnel (CAF) with a 30° surface inclination as well as an alternative version of the CAF with a reduced surface inclination of 10° were tested on the experimental setup. With a straight inclined plate, additional experimental tests on the formation of deposits from the agricultural waste were performed. The straight plate configuration was also used in CFD simulations employed to analyze the impact of the physical properties of the liquid waste on its flow behavior.

Wetting was first tested with the initial CAF concept with a 30° surface inclination; the test was only partially successful, achieving complete liquid wetting of the cone surface. However, liquid separation from the lower edge of the cone and the subsequent vertical fall of the liquid resulted in just minimal or even missing wetting of the underlying funnel. This hindered the complete wetting of the overall CAF system.

It was suggested to modify the initial CAF by increasing the width of its components and reducing their surface inclination to 10°, in order to enhance the wetting and enlarge the surface area of the CAF components. Testing of the modified structure demonstrated complete surface wetting, even at low volumetric flow rates, when prewetting was applied. At higher flow rates, overflow at the outer edge of the funnel was observed. This problem was successfully mitigated using wall wipers.

Experimental investigations of solid deposit formation on a flat inclined plate demonstrated that residue accumulation did not adversely affect the liquid flow behavior. Moreover, the suggested new column internals effectively prevent clogging, which is a problem encountered in columns filled with random packings. The innovative stripping column internals ensure reliable and consistent stripping performance, making air stripping a viable option for reducing the level of ammonia in liquid agricultural waste.

A simulation study was performed to investigate the impact of the contact angle variation on fluid flow and separation. The receding contact angle of 35°, as measured by Bernemann et al.,^15^ was changed to 0° in order to better reproduce the poor dewetting characteristics of the waste observed in experiments. With this new value, the visual similarity between the simulation and the experiment from the previous work was improved and the deviations between measured and simulated *x*-values (horizontal distance the fluid covers after separating from the plate edge) were reduced. In contrast, variation of the static and advancing contact angles reveals only insignificant influence on the wetting of the plate and the liquid separation from the lower edge of the plate.

A further simulation study examined the impact of surface tension and showed that its reduction at moderate volumetric flow rates increases the gas–liquid interface area. Since this effect could not be demonstrated across all flow rates studied, it cannot be reliably exploited to enhance the separation efficiency of the process.

Finally, the influence of viscosity on the flow behavior was investigated by using both measured and estimated viscosities. It was found that a decreased viscosity results in increased *x*-values. As the latter increase the wetted area of the underlying structure, lower viscosities of the liquid waste are beneficial to the process.

Overall, the performed study made a significant contribution toward preventing column clogging from solid deposits through innovative column internals. With the optimized CAF, air stripping can be used for ammonia removal from liquid agricultural waste over long periods.

Future work should be focused on the influence of countercurrent gas flow, which could not be incorporated into the laboratory setup or simulations. Countercurrent gas flow can significantly affect the flow of the liquid waste, especially in its freefall. It is likely that liquid droplets impinge on the surfaces of the internals and the column wall. This would lead to solid deposition in areas not covered by the experiments of this work, e.g., plate backsides. For this reason, additional testing of a demonstrator system is essential. In addition, a demonstrator system would enable separation efficiency assessment of the innovative column internals. Such demonstration experiments are now in the preparation stage. The demonstrator, 10 m in height and with 5 m of internal height, will be used to facilitate the final evaluation of the innovative column internals.

## Latin letters

**f**: volume force, N·m^–3^
**g**: gravity, m·s^–2^
K: consistency index
n: flow behavior index
**n**: unit normal vector
p: pressure, Pa
**S**: viscous stress tensor
t: time, s
**t**: unit tangent vector
**u**: velocity vector, m·s^–1^
x: coordinate, m
y: coordinate, m
z: coordinate, m

## Greek letters

α: volume fraction
γ̇: shear rate, s^–1^
η: dynamic viscosity, Pa·s
Θ: contact angle, °
κ: curvature, m^–1^
ρ: density, kg·m^–3^
σ: surface tension, mN·m^–1^

## Subscripts

A: advancing
cl: contact line
L: liquid
R: receding
S: static
wall: wall boundary
